# Early-Onset Ventilator-Associated Pneumonia in Adults Randomized Clinical Trial: Comparison of 8 versus 15 Days of Antibiotic Treatment

**DOI:** 10.1371/journal.pone.0041290

**Published:** 2012-08-31

**Authors:** Gilles Capellier, Hélène Mockly, Claire Charpentier, Djillali Annane, Gilles Blasco, Thibault Desmettre, Antoine Roch, Christophe Faisy, Joel Cousson, Samuel Limat, Mariette Mercier, Laurent Papazian

**Affiliations:** 1 Réanimation médicale adulte, Pôle Urgences-SAMU-Réanimation CHU, Besancon, Doubs, France; 2 EA 3920, Univ-Fcomte, Besancon, Doubs, France; 3 Pôle pharmaceutique, CHU, Besancon, Doubs, France; 4 EA 4267, Univ-Fcomte, Besancon, Doubs, France; 5 Service de réanimation chirurgicale adulte, Hôpital Central, Nancy, Lorraine, France; 6 Service médico-chirurgical adulte, CHU Raymond Poincaré - Assistance Publique Hôpitaux de Paris, Garches, Ile de France, France; 7 Service de réanimation chirurgicale adulte, CHU, Besancon, Doubs, France; 8 Service de Réanimation médicale - URMITE, Assistance Publique Hôpitaux de Marseille, Marseille, Bouches du Rhône, France; 9 CNRS-UMR 6236, Université de la Méditerranée Aix-Marseille II, Marseille, Bouches du Rhône, France; 10 Service de réanimation médicale adulte, HEGP-Assistance Publique Hôpitaux de Paris, Paris, Ile de France, France; 11 Service de réanimation polyvalente adulte, Hôpital Robert Debré, Reims, Champagne, France; 12 Laboratoire de Biostatistique, CHU, Besancon, Doubs, France; 13 EA3181, Univ-Fcomte, Besancon, Doubs, France; Los Angeles Biomedical Research Institute, United States of America

## Abstract

**Purpose:**

The optimal treatment duration for ventilator-associated pneumonia is based on one study dealing with late-onset of the condition. Shortening the length of antibiotic treatment remains a major prevention factor for the emergence of multiresistant bacteria.

**Objective:**

To demonstrate that 2 different antibiotic treatment durations (8 versus 15 days) are equivalent in terms of clinical cure for early-onset ventilator-associated pneumonia.

**Methods:**

Randomized, prospective, open, multicenter trial carried out from 1998 to 2002.

**Measurements:**

The primary endpoint was the clinical cure rate at day 21. The mortality rate was evaluated on days 21 and 90.

**Results:**

225 patients were included in 13 centers. 191 (84.9%) patients were cured: 92 out of 109 (84.4%) in the 15 day cohort and 99 out of 116 (85.3%) in the 8 day cohort (difference = 0.9%, odds ratio = 0.929). 95% two-sided confidence intervals for difference and odds ratio were [−8.4% to 10.3%] and [0.448 to 1.928] respectively. Taking into account the limits of equivalence (10% for difference and 2.25 for odds ratio), the objective of demonstrative equivalence between the 2 treatment durations was fulfilled. Although the rate of secondary infection was greater in the 8 day than the 15 day cohort, the number of days of antibiotic treatment remained lower in the 8 day cohort. There was no difference in mortality rate between the 2 groups on days 21 and 90.

**Conclusion:**

Our results suggest that an 8-day course of antibiotic therapy is safe for early-onset ventilator-associated pneumonia in intubated patients.

**Trial Registration:**

ClinicalTrials.gov NCT01559753

## Introduction

Pneumonia is the second-most-common nosocomial infection and accounts for around 15% of infections acquired in hospitals. The incidence rate varies from 1% in medical departments to 20% in intensive care units (ICU!) [1]. Intubation and mechanical ventilation (MV) are known risk factors for the acquisition of nosocomial pneumonia [2]. Ventilator-associated pneumonia (VAP) is a common, serious condition and therefore poses a real public health problem, particularly in ICU where the mortality rate for ventilated patients lies above 50% [3], [4]. VAP is defined as having either an early- or a late-onset according to whether it begins before or after the first 5 to 7 days of hospitalization [4]. During early-onset ventilator-associated pneumonia (EOVAP), the normal bacterial flora consists of *Haemophilus influenzae*, *Streptococcus pneumoniae*, methicillin-sensitive *Staphylococcus aureus*, *Moraxella catarrhalis* and non multiresistant *Escherichia coli* [5], [6]. The bacteria isolated may vary due to antibiotic treatment, recent hospitalization and ICU ecology [7], [8], [9], [10]. Several recent studies have evaluated the impact of antibiotic treatment duration on clinical cure and recurrence rates. In a randomized prospective study, Chastre et al. found no clear advantage in prolonging antibiotic treatment to 15 days compared with 8 days when treating late-onset VAP [11]. Other authors [12], [13], [14] have evaluated the use of clinical scores (CPIS) and biological markers (Procalcitonin - PCT) for reducing treatment duration [15]. Resolution of infectious parameters after appropriate antibiotic treatment occurs within 6 days and justifies shorter treatment durations [16]. Although many textbooks do not specify the optimal duration [17], the latest learned society guidelines recommend limiting treatment to 8 days, particularly when appropriate initial antibiotic treatment is administered, and in the absence of isolated nonfermenting gram negative bacilli [18], [19], [20]. Current recommendations are mainly based on one single randomized prospective study related to late-onset VAP [11]. Our randomized, controlled trial comparing 2 antibiotic regimens (8 days versus 15 days) for the treatment of patients with EOVAP who had been ventilated for less than 8 days, was carried out to provide further evidence on the best length of treatment in this ICU population.

Some of the results of these studies have been previously presented [21], [22].

## Methods

The protocol for this trial and supporting consort checklist are available as supporting information; see checklist S1 and protocol S1.

### Inclusion criteria

Patients included in the study were over the age of 18 and had developed EOVAP under invasive mechanical ventilation. The pneumonia was defined as having an early-onset if the patient had been ventilated for more than 24 hours and less than 8 days [23], [24], [25]. A EOVAP diagnosis was considered in the presence of at least 2 or 3 of the following clinical criteria: temperature ≥38.3°C, leukocyte count >10000/mm^3^, excessive purulent or mucopurulent bronchial secretion; and the radiological criterion described by Weinberg [26] (Table 1): increase of 2 points in radiological score compared to chest x-ray on admission, or no improvement of a maximum score (11 or 12) on admission. The diagnosis of pneumonia was confirmed by bronchoalveolar lavage (BAL) culture of ≥10^4^ colony-forming units/ml for at least one bacterial species.

**Table 1:** Radiologic score by Weinberg [26].

By quadrant:
0: normal
1: interstitial infiltrate
2: non-confluent cellular infiltrate
3: condensation
Total: sum scores of each of the 4 quadrants

**Figure 1:**
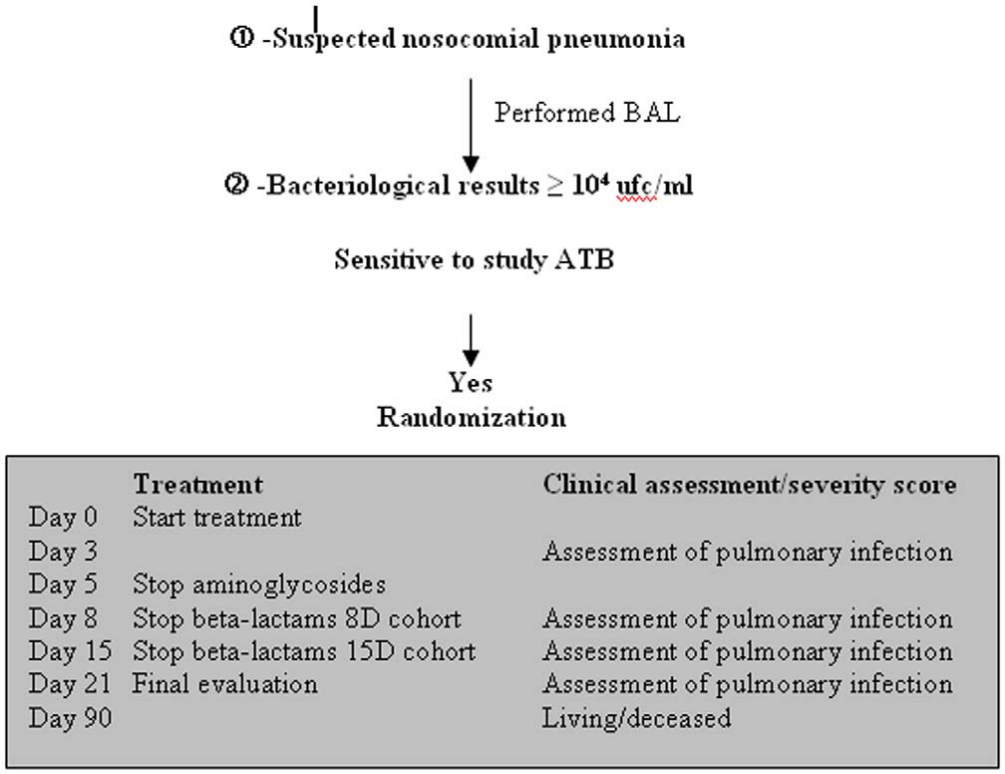
Design of the study governing inclusion of a new patient according to BAL bacteriological results and follow up criteria.

Randomization for treatment duration (8 versus 15 days) was carried out if the bacteria identified in significant concentrations in the BAL samples were susceptible to the authorized antibiotics while for streptococci, a low level resistance to aminoglycosides was not taken into account (Figure 1). Randomization is allocated in each centre by a dedicated randomization table. The assigned treatment arm is communicated by fax at the latest at D5 by the main investigator center.

The exclusion criteria are detailed in table 2. The study protocol had received local ethics committee and CCPPRB (Comite Consultatif de Protection des Personnes dans la Recherche Biomedicale de Franche Comte, number 97/189) approval. Each patient or a member of their family had signed an informed consent form, and written informed consent was obtained from all participants involved in the study.

**Table 2:** Exclusion criteria.

Patients with another source of infection on the day of BAL, except urinary infections with high susceptibility to the study antibiotics
Patients having received curative antibiotics, either following suspected pneumonia or during the 3 days prior to this suspicion, with the exception of surgical antibiotic prophylaxis (defined according to consensus conference) [27]
Patients having received first-line treatment with antibiotics prohibited in this study
Patients with a known allergy to the antibiotics used in the study
Patient participating in another evaluative study
Other exclusion criteria:
-pregnancy,
-cystic fibrosis,
-acquired, induced or congenital immunodeficiency,
-leukopenia <1000 GB/mm^3^,
-neutropenia <500 PN/mm^3^,
-purulent pleurisy or pulmonary abscess at the time of pneumonia diagnosis.

### Treatment initiation

Patients received immediate treatment, according to the standardized severity criteria (Table 3) and direct bacteriological results from BAL samples if available. If immediate antibiotic treatment was initiated, only patients who had been receiving appropriate treatment from the outset were included.

**Table 3:** Indications for antibiotic treatment before bacteriology results.

**Treatment can commence immediately in the presence of at least one of the following organ/system failures combined with sepsis**:
**Respiratory:**
PaO_2_/FiO_2_ <300
**Cardiovascular** (at least one of the following criteria in the absence of hypovolemia):
SAP <90 mmHg or decrease >40 mmHg with signs of peripheral hypoperfusion
Use of inotropic or vasopressor drugs to maintain SAP >90 mmHg
**Renal** (at least one of the following criteria in the absence of chronic renal failure):
Creatinine levels >300 µmol/l
Diuresis <500 ml/24 h or 180 ml/8 h
Patients requiring dialysis
**Central Nervous** (at least one of the following criteria):
Glasgow score <6 (in absence of sedation)
Sudden onset of confusional syndrome
**Hepatic** (at least one of the following criteria):
Bilirubin >100 µmol/l
Alkaline phosphatase >×3 normal level
**Coagulation** (at least one of the following criteria):
Hematocrit 20%
Leukocytosis <2000/mm^3^
Platelets <40 000/mm^3^

All patients included in the study were treated with beta-lactams for 8 or 15 days combined with an aminoglycoside for the first 5 days (Table 4). In the event of a secondary pulmonary infection or the development of extrapulmonary infections, all antibiotics could be used according to the bacteriological data. Once the patient had been included in the protocol, BAL samples were taken for intubated patients prior to making any changes to antibiotic treatment (Table 5).

**Table 4:** Treatments authorized in the study.

**Beta-lactams**:
Amoxicillin plus clavulanic acid (2 g×3/d for 3 days, then 1 g×3/d)
Ceftriaxone (2 g/d for3 days, then 1 g/d)
Cefotaxime (2 g×3/d for 3 days, then 1 g×3/d)
**Aminoglycosides** *:
Tobramycin (loading dose: 6 mg/kg/d, then maintenance dose: 5 mg/kg/d);
Netilmicin (9 mg/kg/d, then maintenance dose 7 mg/kg/d);
Dibekacin (6 mg/kg/d, then maintenance dose 5 mg/kg/d).

* Aminoglycosides were administered in a single dose with a loading dose on the first day of treatment. The dose was adapted in the event of renal failure.

**Table 5:** Definition of secondary infections requiring the initiation of antibiotic treatment for more than 48 hours.

**Bacteremia**:
1 positive hemoculture
or 2 hemocultures positive for *Staphylococcus epidermidis*
**Catheters:**
**Bacteria culture on catheter tip ≥10^3^ cfu/ml**
**Urine:**
Culture ≥10^5^ cfu/ml and >10 PN/field
**Pulmonary:**
Culture ≥10^4^ cfu/ml on BAL
**Others:**
Sinusitis: positive sinus puncture
Puncture sites: positive local samples

Patients were followed up daily during hospitalization in ICU. The onset of intercurrent adverse events associated with the treatment was recorded throughout the follow up (Table 6). All intercurrent infectious events were documented according to the clinical orientation based on appropriate sampling.

**Table 6:** Definition of intercurrent adverse events.

**Definitions**
**Acute renal failure** (creatinemia ×2 compared to pre-treatment value):
Serious: anuria
Persistent diuresis
**Cutaneous complications:**
Serious: Lyell's syndrome
Other
**Digestive complications:**
Serious: pseudomembranous colitis
Other
**Hematologic complications:**
Serious: anemia, leukopenia and/or grade 3 or 4* thrombopenia
Other grades
**Allergic complications**
Serious: anaphylactic shock
Other
**Other (hepatitis, encephalopathy, pneumopathy)**

* World Health Organisation rating.

### Evaluation criteria

#### Primary criteria for evaluating recovery

The primary endpoint of the present study was the clinical cure rate at day 21. Complete clinical recovery was determined by the absence of the following criteria: death, septic shock (except when associated with a documented non-respiratory infection), intercurrent adverse event attributable to the protocol (or for which attributability to the protocol could not be ruled out) requiring modified antibiotic treatment (Table 6), and patients who relapsed. A relapse was defined by a new infectious pulmonary event caused by the same pathogen identified in the initial BAL fluid (regardless of its susceptibility profile), associated with clinical and radiological signs of nosocomial pneumonia (occurring ≥4 days of treatment) or a worsening of 2 points of the baseline SOFA score.

#### Secondary criteria

The study focused on establishing the determinants of clinical response on day 21: incidence of secondary nosocomial infections; number of patients on antibiotic treatment; total number of days of antibiotic treatment; duration of MV; number of patients still under ventilation; number of patients still in ICU; length of stay in ICU on day 21; and mortality rate at 3 months. The following set of complementary results enabled a better characterization of the study cohorts: isolated pathogens; frequency of positive hemocultures; and proportion of patients receiving first-line or secondary treatment (Table 3).

### Statistical analysis

The trial was designed to show equivalence in the clinical response rates between the 2 treatment groups. A sample size of 220 patients was determined to be adequate to demonstrate equivalence with a power of 80% and a basal response rate of 90%. The limits of the 95% confidence interval (CI) for the difference between the response rates in the 2 treatments groups were calculated, and the equivalence accepted if it did not exceed 10%.

Comparisons of baseline patient characteristics and secondary criteria between the 2 cohorts were performed using the t-test for continuous variables and the chi-square test or Fisher's exact test for categorical variables.

The efficacy analysis was conducted using an intent-to-treat approach. Difference (d) and odds-ratio (OR) of response rates and their 95% CIs were used to test the equivalence between the 2 cohorts [28], [29].

All P values were two-sided. Data are presented as mean values with the standard deviation in brackets for normal distribution variables, and as median and quartile values for others (SAS institute inc. USA version 9.1).

## Results

### Patient characteristics

**Figure 2:**
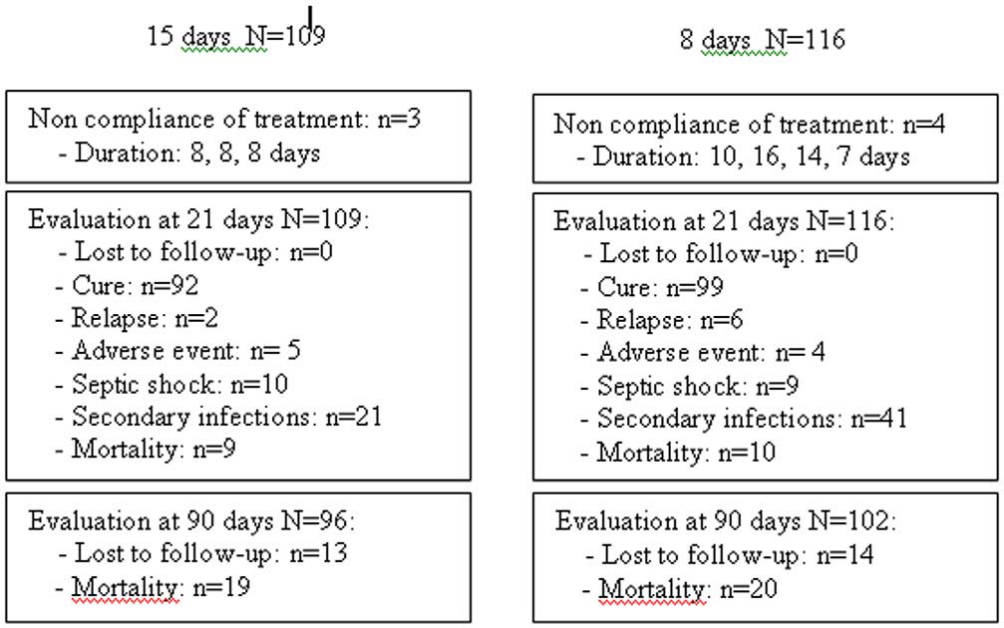
Data regarding number of patients included, treated according to protocol and followed up at 21 and 90 days.

225 patients (109 in the 15 d cohort and 116 in the 8 d cohort) were included and evaluated in 13 different centers over a period of 4 years from 1998 to 2002, and 6 of the centers recruited 96% of patients (figure 2). The patients' baseline characteristics were comparable in the 2 groups (Table 7). The cause of coma was traumatic in 38.9% and 40.5% of patients in the 15 d and 8 d cohorts respectively.

**Table 7:** Admission characteristics of the study patients according to the duration of antibiotic treatment.

	15 days	8 days	
	N = 109	N = 116	
	n (%)	n (%)	
	m± sd	m± sd	p Values
**Sex-ratio**	2.4	2.4	0.99
**Age** (years)	48.2±19.5	49.6±19.6	0.59
**Weight** (kg)	73.1±16.4	73.6±17.0	0.80
**Origin**			
-hospital	33 (30.3)	35 (30.2)	0.99
-home	76 (69.7)	81 (69.8)	
**Reason for admission**			
-medical	63 (57.8)	59 (50.9)	0. 35
-surgical (scheduled)	2 (1.8)	0	0.23
-surgical (emergency)	6 (5.5)	12(10.3)	0.22
-post-trauma	42 (38.5)	47(40.5)	0.22
**Coma**			
-RTA*	42 (38.9)	47 (40.5)	0.89
-toxic	11 (10.2)	6 (5.2)	0.21
**SAPS** † **II**	39.7±12.5	39.2±13.4	0.76
**Delay between:**			
**-hospital and ICU** ‡ **admission**			
0 day	76 (69.7)	79 (68.1)	0.82
1<D<3 days	7 (6.4)	10 (8.6)	
more than 4 days	26 (23.9)	27 (23.3)	
**-hospital admission and intubation** (days)	1.0±2.9	2.1±8.9	0.22
Median	0	0	
IQR	1	1	
**-ICU admission and intubation** (days)	0.13±1.4	0.49±2.8	0.22
Median	0	0	
IQR	0	0	
**-Intubation and BAL** § (days)	3.4±1.8	3.5±1.9	0.59
Median	3	3	
IQR	2	3	
**Clinical Criteria for Pneumonia**			
-Fever	98 (89.9)	103 (88.8)	0.79
-Leukocytosis	69 (63.3)	78 (67.2)	0.54
-Purulent secretion	108 (99.1)	113 (97.4)	0.62
-Radiologic score	4.36 (1.9)	4.55 (2.1)	0.48
**Treatment**			
**Beta-lactam**			
**-**Amoxicillin+Clav. Ac.	39 (35.8)	48 (41.4)	0.41
-C3G**	70 (64.2)	68 (58.6)	
**Aminoglycoside**			
-Netilmicin	35 (32.1)	37 (31.9)	0.97
-Tobramycin	74 (67.9)	79 (68.1)	

Abbreviations:

* RTA: road traffic accident;

† SAPS: simplified acute physiological score;

‡ ICU: intensive care unit;

§ BAL: bronchoalveolar lavage;

** 3GC: third generation cephalosporin.

### Bacteriology

There were no significant differences between the proportions of gram-negative bacilli (p = 0.32) and gram-positive cocci (p = 0.65) from one group to the other. 32.9% of the pathogens isolated in BAL samples were MSSA in the 15 d cohort, and 28.2% in the 8 d cohort. The samples were multimicrobial for 54.1% of patients in the 15 d cohort and 37.9% of patients in the 8 d cohort (p = 0.03), but the antibiotics used were active on all pathogens. The bacteria isolated in the BAL samples are shown in Table 8. For 2 (15 d cohort) and 3 (8 d cohort) patients, several strains of MSSA were isolated in the BAL fluid. One MSSA pathogen was identified in the BAL samples of 58 patients in the 15 d cohort and 45 patients in the 8 d cohort (p = 0.35).

**Table 8:** Microbiology of pneumonia according to the treatment group.

	15 days*	8 days*	
	N = 182	N = 170	
	n (%)	n (%)	p Values
**Gram+ cocci**	**104 (57%)**	**93 (54.7%)**	0.65
Staphylococcus	60	48	0.34
-MSSA	57	46	
-MSCNS	3	2	
Streptococcus	44	45	
-Pneumoniae	22	23	
-Other	21	22	
-Enterococcus	1	0	
**Gram− cocci**	**4**	**2**	
-Neisseria	3	2	
-Other	1	0	
**Gram+ bacilli**	**4**	**1**	
-Corynebacterium	4	1	
**Gram− bacilli**	**70**	**73**	0.32
Enterobacteria	23	20	0.46
-Escherichia coli	12	10	
-Proteus	6	1	
-Serratia	1	0	
-Citrobacter	0	3	
-Klebsiella	2	4	
-Enterobacter	2	1	
-Hafnia	0	1	
Haemophilus	43	52	0.41
Chryseomonas	1	0	
Branhamella	2	0	
Moraxella	0	1	
Pasteurella	1	0	
**Anaerobes**	**0**	**1**	

* The number of bacteria isolated in the BAL sample is >1 for 59 patients in the 15 days and 44 patients in the 8 days cohorts.

18 patients (8 in the 15 d cohort, 10 in the 8 d cohort) presented at least one positive hemoculture during the 24 hours of BAL sampling. For 7 of these patients, the isolated pathogen in the hemoculture was the same as in the BAL fluid. We found 5 MSSA, 1 *pneumococcus* and 1 *Escherichia coli*. For the remaining 11 patients, the pathogen was not isolated in the BAL fluid or in an another focus of infection. We found 6 *Staphylococcus epidermidis* or negative coagulase, 1 *enterococcus*, 3 *streptococci* and 1 MSSA. The mortality rate for patients with positive hemoculture did not differ from the overall study population (p = 0.82; Fischer's exact test).

### Treatment duration and compliance

Immediate treatment was initiated following BAL in 77 (71%) and 81 (70%) patients in the 15 d and 8 d cohorts respectively. The mean durations for first-line treatment were 13.1 (3.6) days and 7.8 (1.6) days for the 15 d and 8 d cohorts respectively (p<0.01).

### Principal criteria

According to our judgment criteria, a total of 191 out of the 225 patients (84.9%) included in the study were cured; 92 out of 109 (84.4%) in the 15 d cohort and 99 out of 116 (85.3%) in the 8 d cohort (d = 0.9%, OR = 0.929). 95% two-sided CIs for difference and OR were [−8.4% to 10.3%] and [0.448 to 1.928] respectively. Taking into account the limits of equivalence (10% for difference and 2.25 for OR), the protocol objective of demonstrating equivalence between the 2 treatment regimens was fulfilled.

### Secondary objectives

The mortality rate evaluated on day 21 was comparable in the 2 cohorts. 19 patients died, 9 (8.3%) in the 15 d cohort and 10 (8.6%) in the 8 d cohort (p = 0.92). The median delay between antibiotic treatment initiation and death was also comparable in the 2 cohorts; 11.8 (5.7) days and 10.7 (4.8) days for the 15 d and 8 d cohorts respectively (p = 0.66). At 3 months, 88% of all patients had been evaluated. Out of 96 patients in the 15 d and 102 patients in the 8 d cohort, the mortality rate was almost identical: 17.4% and 17.2% respectively (p = 0.99).

Nine patients experienced an adverse event requiring treatment modification: 5 in the 15 d and 4 in the 8 d cohort. The cause of the event was cutaneous in 4 cases, digestive (*C. difficile*) in 3 cases and hepatic in 2 cases. 19 patients experienced septic shock (10 in the 15 d and 9 in the 8 d groups), 17 in the absence of new infectious event, and 2 in the presence of an undocumented infectious intercurrent event.

Eight patients experienced a relapse of the initial pulmonary infection with isolation of the same pathogen in pulmonary samples; 2 in the 15 d and 6 in the 8 d cohort (NS). One *pneumococcus* and 1 methicillin-resistant *Staphylococcus aureus* (MRSA) pathogen (initial presence of 1 MSSA pathogen) persisted in the 15 d cohort. One *Haemophilus*, 1 *Serratia*, 3 MSSA and 1 MRSA (initial presence of 1 MSSA) persisted in the 8 d cohort. For both durations, the median delay between treatment initiation and relapse diagnosis was 11.6 days (min = 7; max = 18).

### Secondary nosocomial infections during the 21 days after randomization

A secondary infection was diagnosed in 19.3% of patients in the 15 d cohort and 35.3% of patients in the 8 d cohort (p<0.01) (Table 9). Comparisons between patients with and without secondary infections are depicted in table 10. Patients presented more pulmonary infections than extrapulmonary infections. 116 BAL were performed during the follow-up. 71 BAL done after initiation of the treatment were negative (26 for the 15 days and 45 for the 8 days arms, p = 0.01). There was no difference in the proportion of patients with a negative BAL, respectively 24 (22.0%) and 33 (28.7%) for the 15 and 8 days groups (p = 0.27). The isolated bacteria are reported in table 11. In patients in whom antibiotics were not started immediately, we also did not find any mortality difference at day 21. We compared the superinfection rate in patients receiving either a third generation cephalosporin or amoxicilline-clavulanic acid. Overall, there was no difference (respectively 28% and 26.4%). We analysed this superinfection rate according to the length of treatment and to the type of antibiotic (third generation cephalosporin or amoxicilline-clavulanic acid) and did not find any significant difference (15 days group respectively 20% and 18%; 8 days group respectively 37% and 33%).

**Table 9:** Patients with documented secondary infections.

	15 days	8 days	p Value
	N = 109	N = 116	**
**Patient,** n (%)	21(19.3)	41(35.3)	<0.01
**Infections,** n (%)	22 (20.2)	46 (39.7)	<0.01
Origin Pulmonary, n (%)	15 (13.8)	30 (25.8)	0.03
Sensitive to first-line treatment			
**-S** * **bacteria**, n(%)	12 (54.5)	28 (60,8)	0.76
**-R** † **bacteria**, n(%)	10 (45.5)	18 (39.2)	
**Secondary infection delay occurrence** (day)	11.6±5.6	10.5±4.9	0.57

Abbreviations:

* sensitive,

† resistant,

** Fischer exact test.

**Table 10:** Comparisons between patients with and without secondary infections.

	All population			15 days			8 days		
	N = 225			N = 109			N = 116		
	n (%)			n (%)			n (%)		
	m± sd			m± sd			m± sd		
	S	NoS	p	S	NoS	p	S	NoS	p
	N = 62	N = 163	Values	N = 21	N = 88	Values	N = 41	N = 75	Values
**Origin**									
-hospital	22(35.5)	46(28.2)	0.29	7(33.4)	26(29.6)	0.74	15(36.6)	20(26.7)	0.27
-home	40(64.5)	117(71.8)		14(66.7)	62(70.5)		26(63.4)	55(73.3)	
**Reason for admission**									
-medical	32(53.3)	90(55.2)	0.80	11(57.9)	52(59.1)	0.92	21(51.2)	38(50.7)	0.95
-surgical (scheduled)	0(0.0)	2(1.2)	1	0(0.0)	2(2.3)	1	0(0.0)	0(0.0)	
-surgical (emergency)	6(9.84)	11(6.75)	0.41	1(5.0)	4(4.55)	1	5(12.2)	7(9.3)	0.75
-post-trauma	26(42.6)	63(38.7)	0.59	9(45.0)	33(37.5)	0.53	17(41.5)	30(40.0)	0.88
**Coma toxic**	1(1.6)	16(9.82)	0.05	1(5.0)	10(11.4)	0.69	0(0.0)	6(8.0)	0.09
**SAPS** † **II**	38.5±13.0	39.8±13.0	0.50	44.5±10.8	38.5±12.7	0.05	35.4±12.9	41.2±13.3	0.02
**Delay between**									
-hospital and ICU‡ admission (days)	0,9±1.8	1.4±7.1	0.35	0.8±2.2	0.9±2.6	0.83	0.9±1.6	2±10	0.35
-hospital admission and intubation (days)	1.2±3.6	1.7±7.2	0.45	1.0±4.1	1.1±2.6	0.99	1.3±3.4	2.6±10.2	0.32
-ICU admission and intubation (days)	0.3±3.1	0.3±1.9	0.98	0.2±2.2	0.1±1.3	0.73	0.4±3.5	0.5±2.4	0.77
-Intubation and BAL§ (days)	3.7±1.7	3.4±1.9	0.27	3.7±1.9	3.3±1.7	0.40	3.7±1.5	3.4±2.1	0.49
**Clinical Criteria for Pneumonia**									
-Fever	53(85.5)	148(90.8)	0.25	19(90.5)	79(9.8)	1	34(82.9)	69(92.0)	0.22
-Leukocytosis	41(66.1)	106(65.0)	0.88	15(71.4)	54(61.4)	0.39	26(63.4)	52(69.3)	0.52
-Purulent secretion	60(96.8)	161(98.8)	0.30	20(95.2)	100(100.0)	0.19	40(97.6)	73(97.3)	1
-Radiologic score	4.8±2.5	4.3±1.8	0.25	4.8±2.5	4.3±1.7	0.37	4.7±2.5	4.5±1.8	0.54
**Treatment**									
**Beta-lactam**									
-Amox.+Clav.Ac.	23(37.1)	64(39.3)	0.77	7(33.3)	32(36.4)	0.79	16(39.0)	32(42.7)	0.70
-C3G*	39(62.9)	99(60.7)		14(66.7)	56(63.6)		25(61.0)	43(57.3)	
**Aminoglycoside**									
-Netilmicin	19(30.7)	53(32.5)	0.79	6(28.6)	29(33.0)	0.7	13(31.7)	24(32.0)	0.97
-Tobramycin	43(69.4)	110(67.5)		15(71.4)	59(67.1)		28(68.3)	51(68.0)	
**First-line treatment**	9.0±3	10.9±3.9	<0.01	11.7±3.8	13.5±3.5	0.02	7.7±1.1	7.9±1.8	0.36
**Antibiotic treatment** **	15.8±4.7	12.3±4.1	<0.01	18.9±2.6	14.5±3.0	<0.01	14.3±4.8	9.7±3.7	<0.01
**Mortality 21 days**	4(6.5)	15(9.2)	0.51	0(0.0)	9(10.2)	0.20	4(9.8)	6(8.0)	0.74

Abbreviations:

† SAPS: simplified acute physiological score;

‡ ICU: intensive care unit;

§ BAL: bronchoalveolar lavage;

* 3GC: third generation cephalosporin;

** all molecules grouped together.

**Table 11:** Microbiology of secondary infections.

	Urinary		Pulmonary		Bacteremia		Catheter		Sinus		Other	
	infections		infections				infections		infections			
	15 d–8 d		15 d–8 d		15 d–8 d		15 d–8 d		15 d–8 d		15 d–8 d	
**Gram+ cocci**												
-MSSA				4								1**
-MRSA			1	3	1							
-MSCNS				2								1*
-MRCNS				1		2	1		1			
-Streptococcus												
Pneumoniae			1‡‡									
Other			1	2						1	1‡	
Enterococci	1	1								1		
**Gram− cocci**												
-Neisseria				1								
**Gram+ bacilli**												
-Corynebacterium	1§§											
**Gram− bacilli**												
-Escherichia coli	1		1	3						3	1‡	
-ESBL§ Escherichia coli		1										
-Proteus				1				1				
-Serratia			2	2								
-ESBL§ Serratia				1								1**
-Citrobacter				1								
-ESBL§ Enterobacter			1	1								
-Haemophilus				5								
-Acinetobacter		1	3	4					1			
-CAZ-S^†^ Pseudomonas		1	3	6				1				1**
-CAZ-R^††^ Pseudomonas	1		2	3						1		
-Aeromonas			1									
**Anaerobes**												
-Prevotella										1		
**Candidas**			1	2								

* CSF,

‡ pleural fluid,

** chest drain,

§ ESBL (Extended-Spectrum Beta-Lactamase),

CAZ-S^†^ (sensitive to ceftazidime),

CAZ-R^††^ (resistant to ceftazidime),

‡‡ Reduced sensitivity to beta-lactams and high-level aminoglycoside resistance,

§§ Sensitive to chloramphenicol, cyclins, vancomycin, teicoplanin.

Taking into consideration the onset of a secondary infection as an added failure criterion, the percentages of cured patients (clinical success) were 64.2% in the 15 d cohort and 49.1% in the 8 d cohort (d = 15.1%). 95% two-sided CI for difference was [2.3% to 27.9%]. Taking into account the limit of equivalence (10%), equivalence between the 2 treatments was rejected.

### Other secondary criteria

Sixty-four patients in the 15 d cohort (58.7%) and 66 (56.9%) in the 8 d cohort had been discharged alive from the ICU by day 21. The mean duration of hospitalization in ICU was 15.7 (5) days in the 15 d cohort and 15.9 (5.1) days in the 8 d cohort (p = 0.82). The length of stay in ICU after treatment initiation was 11.9 (4.4) days in the 15 d cohort and 11.3 (3.7) days in the 8 d cohort (p = 0.42). The mean duration of intubation in ICU was 13.4 (5.9) days in the 15 d cohort and 13.6 (5.3) days in the 8 d cohort (p = 0.81). The number of patients intubated at day 21 did not differ between the 2 groups (36 in the 15 d cohort and 40 in the 8 d cohort; p = 0.88).

The mean duration of antibiotic treatment (all molecules grouped together) during the 21 days of follow up was 15.4 (3.4) days for the 15 d and 11.3 (4.7) days for the 8 d cohort (p<0.01). The median durations of antibiotic treatment during the 21 days of follow up were 15 and 8 days respectively. The number of patients still on antibiotics on day 21 was significantly different between both groups; 20 patients in the 15 d cohort and 39 in the 8 d cohort (p = 0.01). Among these patients, 13 in the 15 d cohort and 18 in the 8 d cohort had left ICU (p = 0.41).

## Discussion

The results of this prospective, randomized study showed that 15 and 8 days of initially appropriate antibiotic treatment for EOVAP were equivalent according to the rate of complete clinical recovery. Additionally, 21 and 90-day mortality rates as well as durations of mechanical ventilation and of ICU stay were not different between both strategies. However, the rate of secondary infections was higher in the 8 d cohort than in the 15 d cohort, resulting in a lower rate of clinical cure in the 8 d strategy when including the occurrence of a secondary infection [11]. Despite this difference, the number of days of antibiotic treatment was lower in the 8 d cohort and around 50% of the secondary infections remained sensitive to the first-line treatment.

In the present study, VAP was defined as early when it occurred between 1 and 8 days after initiation of mechanical ventilation. Moreover, antibiotic choice was protocolized using quite limited-spectrum antibiotics and appropriate first-line antibiotics were a pre-requisite for patient inclusion in our study. Compared to our definition, early onset pneumonia has been commonly defined as occurring in the first 4 days. However, extended periods of 5 to 7 days have been used in the literature [9], [23], [24], [25], [31]. To note, 94% of our patients were included in the 6 days following initiation of mechanical ventilation. The bacteriology of EOVAP rarely consists of multiresistant pathogens. Microbiological data from BAL fluid of our patients confirmed this with a strong predominance of MSSA, streptococci, Haemophilus and enterobacteriacae species. Although the isolation of resistant bacteria has been reported in EOVAP [8], [9], [32], the percentage of resistant bacteria only accounted for 13% of those isolated in a recent study [7], and a strategy using limited-spectrum antibiotics allowed for appropriate treatment in 80% of cases of EOVAP [33], [34]. To note, polymicrobial samples were more common in our 15 d cohort. The reason for this is not clear, but the sensitivity of the bacteria to the initial treatment means that it probably had no real impact. As for all our patients, a polymicrobial incidence of around 50% was reported during VAP. Combes et al found that the epidemiology and outcomes of patients with monomicrobial and polymicrobial VAP did not differ significantly [35]. In our study, polymicrobial samples were not detrimental to recovery.

Until recently, recommendations for treatment duration of VAP were based only on the advice of experts [4], [18], [30] as there was no prospective, randomized study available when the present trial was initiated. Chastre et al. found no clear advantage in prolonging antibiotic treatment to 15 days compared with 8 days when treating late-onset VAP [11]. Our data strongly suggest that a 8-day treatment of EOVAP enables recovery in the same proportions as a 15-day regimen using the same clinical and microbiological cure criteria than those used in other studies [36], [37], [38]. Equivalence was obtained according to the criteria that we defined at first, in particular that the onset of a secondary pulmonary infection did not exclude the possibility that the primary infection had been cured. These data confirm recommendations for reducing treatment to 7–8 days for EOVAP [4], [12], [16], [39]. Moreover, the reception of negative microbiological samples [16], as well as the clinical response evaluated by composite scores [12], [40] may help to securely shorten the duration of antibiotic treatment of VAP. Recently, the implementation of an antibiotic protocol for VAP including these tools has indeed been associated with a reduction of antibiotic treatment duration from 10.1 (8) to 6.2 (3.3) days associated with a reduction in the duration of mechanical ventilation [31]. These objectives have now been extended to early- and late-onset pneumonia by learned societies [18], [19], [20] except if the etiologic agent was *P. aeruginosa* or an *Acinetobacter* species which carry a greater risk of relapse. Prolonged antibiotic therapy may also be justifiable in cases of pulmonary infections caused by MRSA due to the delayed response to treatment [41], and the increased risk of relapse [42]. Further research is required to determine if a 8-day treatment of resistant bacteria EOVAP is safe since these cases have been excluded from the present study.

In our study, the rate of secondary pulmonary and extrapulmonary infections was significantly higher in the 8 d cohort. The risk of acquiring a nosocomial infection is high during the first few days following admission to ICU [43], [44], and patients frequently develop several infections simultaneously or close together [44], [45]. An early but short treatment of VAP could therefore lead to a higher rate of infections rapidly unmasked after antibiotic stop or treated too shortly. Indeed, a majority of superinfections in the 8d cohort occurred in the few days following the end of antibiotic treatment. In the multicenter trial of 8 vs 15 days of antibiotic treatment for late onset VAP by Chastre et al [11], neither relapses nor superinfection rates were different between both strategies. Several reasons related to the type of infection and to their treatment may explain this difference. First, whereas in this latter study, over 80% of patients had received curative antibiotics in the 15 days prior to inclusion, this situation did not apply to any of our patients. Moreover, broad spectrum antibiotics were used in the Chastre et al [11] study according to the isolated bacteria. Finally, a combination of antibiotics including an aminoglycoside was used in more than 90% on day 1 and was still administered on day 8 in more than 30% of patients while in our study, we protocolized the use of narrow spectrum antibiotics and aminoglycosides were stopped on day 5.

We did not recommend using fluoroquinolones and proposed an aminoglycoside treatment with a limited duration, identical in the 2 cohorts. Nevertheless, the use of combination therapy is debatable considering the bacteria expected in EOVAP. Indeed, a recent trial suggested that monotherapy is associated with similar outcomes compared with combination therapy in patients who have suspected late VAP and who are at low risk for difficult-to-treat gram-negative bacteria, [46]. This strategy would probably be applicable to our patients, excepted in patients with septic shock.

Several limitations were apparent in our study. First, although it was planned, it has not been possible to accurately quantify the number of patients who were not included in the study, as well as the microbiologic characteristics of their VAP. Nevertheless, the non-inclusion of the most critically-ill patients cannot be the main reason. Indeed, patients with a deep source of infection (abscess or purulent pleurisy) as well as immunodeficient patients were excluded from the study. Moreover, mean SAPS II was 39, 70% of the study population had 2 organ failures and 18 patients presented with bacteriemia. Second, except for mortality rate, follow-up was limited to 21 days. However, only 31% of patients were still hospitalized on day 21, what limits the interest and validity of a longer follow-up. Third, although this study enrolled patients in 13 centers, 6 of them included 96% of patients. However, 5 patients were included in low inclusion rate centers in each arm. Moreover, randomisation was processed by center to avoid any center effect. Fourth, patients were treated with a combined therapy. While the aminoglycosides are not great pneumonia drugs, they clearly have some benefit resulting in a synergistic effect with beta-lactams and a larger antibiotic spectrum. As a consequence, aminoglycosides might have skewed the results toward equivalence.

In conclusion, our study enabled us to recommend reduced treatment duration of 8 days for early-onset VAP, while respecting the contra indications. The higher rate of secondary infections in the 8 days than in the 15 days treatment strategy highlights the importance of rigorous clinical monitoring and the possible interest of biological markers for securing this strategy.

## Supporting Information

Protocol S1 **Trial protocol.**
(DOC)

Checklist S1 **CONSORT checklist.**
(DOC)
