# Supplementary material for: Early-Onset Ventilator-Associated Pneumonia in Adults Randomized Clinical Trial: Comparison of 8 versus 15 Days of Antibiotic Treatment
Source: PLoS One. 2012 Aug 31;7(8):e41290. doi: 10.1371/journal.pone.0041290 (PMC3432026; doi:10.1371/journal.pone.0041290)
Supplement: Protocol S1 — Trial protocol. (DOC) [file pone.0041290.s001.doc]

**Index**

**1. Introduction**....................................................................................... .......... **p 2**

**2. Rational**............. ....................................................................... **p 3**

**3. Aims of the study**........................................................................ .............. **p 5**

**4. Diagnosis of early onset nosocomial pneumonia** ............................. **p 6**

**5. Treatments**.................................................................................................. **p 7**

**6. Patients**......................................................................................................... **p 9**

**7. Evaluation criteria ……….**.............................. **p 11**

**8. Timing**................................................................................ …**p 14**

**9. Organisation**...................................................................... …**p 16**

**10. Secondary aims**.............................................................................. . **p 18**

**11. Statistical analysis**......................................................................  **..p 21**

**12. Results**.............................................................................. ..**p 23**

**Appendices**..................................................................................... … **p 24**

**References……**...............................................................................................**p 41**

1. Introduction

Nosocomial Pneumonia is defined as an infection of the lung parenchyma acquired in the hospital, which means that it was neither present nor in incubation at admission (criteria defined by the Center for Disease Control) (12). Nosocomial pneumonia is the third most prevalent cause of nosocomial infection, representing about 15% of infections acquired in hospital (17). Their prevalence among patients varies between 1% and 20% respectively in medical and critical care units (8). Intubation and mechanical ventilation are recognized risk factors for the acquisition of nosocomial pneumonia (15). Among all nosocomial infections, nosocomial pneumonia is associated with the highest mortality rate. Mortality is higher than 50% in ventilated patients (9). Their frequency and severity, pose a real public health problem, particularly in intensive care units.

Despite the difficulty of accurately assessing morbidity and the additional cost, some studies clearly show that nosocomial pneumonia is at the origin of complications (septic shock, infections...) and results in an extension of stay in many ventilated patients (9.16)

2. Rational of the study

Early onset nosocomial pneumonia will be defined by a new occurrence of pulmonary infection on mechanical ventilation in the first 8 days of ventilation and on the profile of antibiotic susceptibility of the microorganism (sensitivity retained to 3rd generation cephalosporins and aminoglycosides). In early onset nosocomial pulmonary infection, bacterial flora normally found consists of Haemophilus Influenzae, Streptococcus Pneumoniae, Staphylococcus Aureus, Methicillin-sensitive, Moraxella Catarrhalis, Escherichia Coli (1,7,24).

This study of early nosocomial pulmonary infection will likely restrict findings to "community", non-multi drug resistant microorganisms and patients without multiple infections. This option is available to obtain homogeneous groups from the infectious point of view and to standardize the evaluation criteria.

**2.2 Duration of treatment**

As a general rule, the duration of antibiotic treatment of pulmonary infection is rarely addressed, except for a few specific pathologies (e.g. Mycobacterium tuberculosis). It is interesting to note that the different consensus of Infectious Pathology conferences and pharmacological and microbiological textbooks regularly conflict over the duration of the anti-infective treatment (19). The duration of antibiotic treatment is still empirical, available data is fragmentary and studies that investigate antibiotic therapy are not specifically exploring the duration of the treatment. The duration of treatment of community acquired pulmonary infection varies between 5 and 14 days according to the author choice (22), or even 3 days using newer drugs with longer half-lives (2). For nosocomial pulmonary infection, treatment durations are not standardized (5). It is simply mentioned the concept of "usual" treatment of at least 15 days. However, recent studies used 10 days of treatment without significant decrease in disease resolution compared to usual treatment (6,10,31).

It is essential to clarify the optimal duration of antibiotic treatment. Indeed, any excessive extension of treatment may increase the occurrence of adverse effects (renal toxicities, hepatic...), and induce resistance of bacteria to antibiotics (selection pressure), colonization of the patient by multi-resistant bacteria and an increase in cost of treatment (13).

**2.3 Treatment modalities**

Some studies show the possibility of treating pulmonary nosocomial disease with monotherapy, particularly with the newer drugs (10.18). However, most authors agree that it is still preferable to use a combination of antibiotics in the treatment of nosocomial pulmonary disease (1). This therapeutic attitude aims to broadening the antibiotic spectrum (especially when intitiating the treatment), and provide a synergystic effect in order to accelerate the bactericidal effects and decrease the emergence of resistant microrganisms. The concurrent use of beta lactams and aminoglycosides is justified as we excluded the new broad spectrum antibiotics from the protocal.

The use of high dose beta-lactam antibiotics is justified by the existence of an important bacterial load in nosocomial pulmonary infection (26) and by the changes (increase in the volume of distribution) pharmakokinetic observed in the resuscitated patient (3). High dose antibiotic therapy in the first 3 days, was recently being described in the treatment of community pulmonary infection requiring hospitalization (4).

The rational for once daily administration of aminoglycosides is multiple: clinical efficacy, toxicity, gain of nursing time and potential reduction of costs (20). All clinical studies show an equivalent efficiency for the three modes of administration of aminoglycosides (1, 2 or 3 injections per day), without change of the level of toxicity (14). Some authors (29) observe a better renal tolerance of the netilmicine administered in a single dose. The pharmacokinetic study of the aminoglycoside antibiotics administered in a single dose in the critical care patient highlights a decrease of 15 to 35% of the peak plasma concentration, compared to that observed in healthy volunteers (21). This phenomenon is due to the increase in the volume of distribution (3). These arguments support the case for a higher loading dose of 25-30% (28) and an increase of the daily dose when injected as a single daily dose.

**3. Objectives of the study**

**This is a prospective, randomized, open, multi-center study.**

**3.1 Primary Objective**

To show that antibiotic therapy of 8 or 15 days is equivalent in terms of clinical cure rates in the treatment of early onset nosocomial pulmonary infection in patients receiving mechanical ventilation.

- 1. **Secondary Objective**

**. Study of nosocomial infections:** assessing the influence of the duration of antibiotic treatment on the rate of potentially fatal pulmonary and extra-pulmonary infection (definition of nosocomial infections: annex XIII).

- **Study of bacterial Ecology:** research of changes in the flora of the gut under treatment and analysis of pathological samples (antibiotic resistance phenotypes +/- genotypes).

**. Economic Survey:** analyze the costs related to the treatment of nosocomial pneumonia and its complications in the two study groups.

**4 Diagnosis of early onset nosocomial infection**

The diagnosis of early onset ventilator associated pneumonia is confirmed if all of the following criteria is met:

**4.1 Clinical signs**

- artificial ventilation started since at least 24 hours and for less than 8 days

- 2 of 3 following criteria: temperature (>38.5 ° C or (<36 ° C), leukocytosis (>12 000 / mm^3^ or (<5 000 / mm^3^), abundant, purulent or mucopurulent sputum

- absence of another focus of infection documented the day of the BAL

**4.2 Radiological criteria**

- increase of 2 points in the radiological score from chest radiography done on admission (annex I)

**4.3 Bacteriological results**

- quantitative culture of bronchoalveolar lavage (BAL) greater than 104 cfu / ml (Appendix II)

**5. Treatment**

**5.1 Intitiating the treatment**

A BAL will be made upon suspicion of nosocomial pneumonia (clinical and radiological criteria defined in Section 4). Patients will be treated either after the BAL bacteriological results, or immediately after the BAL according to the predefined severity criteria (Appendix III). If the antibiotic treatment is started immediately after the BAL, the patient will be included in the study as long as the treatment is not changed after the results of the sensitivity.

**5.2 Treatment**

All patients included in the study will be treated by a combination of antibiotics during the first 5 days (Beta-lactam + Aminoglycoside), then by monotherapy (Beta-lactam alone) for 3 or 10 days according to the group. The beta-lactam antibiotics will be administered in high doses during the first 3 days of treatment. Aminoglycosides will be administered as a single daily dose, with a loading dose administered on the first day of treatment. The patient will remain hospitalized (intensive care units or other units) for the duration of the treatment.

Definition :

- J0 = introduction of treatment whatever the time is
- J1 = first full day of treatment
- J4 = decrease in the dose of beta-lactam antibiotics, after 3 full days of treatment with high dose antibiotics; intermediate evaluation will be performed
- J6 = Stop aminoglycosides, after 5 full days of treatment
- J9 = Stop beta-lactam antibiotics in the "short" arm.
- J16 = Stop of the beta-lactam antibiotics in the "long" arm.
- J21 = Final evaluation.

**5.3** **Authorized antibiotics in the study**

**- Beta-Lactams**

- Amoxicillin + clavulanic Acid : **2 g TDS for 3 days, then 1 g TDS**
- Keftriaxone : **2 g OD during 3 days, then 1 g OD**
- Kefotaxime : **2 g TDS during 3 days, then 1 g TDS**

**- Aminoglycosides**

- Tobramycin : **loading dose of 6 mg/kg OD, then** **5 mg/kg OD (adaptation in case of renal failure)**
- Netilmicin : **loading dose of 10 mg/kg OD, then** **8 mg/kg OD (adaptation in case of renal failure)**
- Dibekacin : **loading dose of 6 mg/kg OD, then** **5 mg/kg OD (adaptation in case of renal failure)**

**5.4** **Non-authorized antibiotics in the study**

All other antibiotics, outside those proposed in the protocol, will not be used in the initial treatment.

**5.5** **Antibiotic adjustment**

If a new pulmonary or non pulmonary superinfection develops, all antibiotics are usable, supported by bacteriological data. Any change of antibiotic treatment for new pulmonary infection occurring after the beginning of the protocol has to be supported by a BAL if the patient is intubated.

**6. Patients**

**6.1 Inclusion criteria**

- Early onset nosocomial pneumonia (delay in onset is less than or equal to 8 d of artificial ventilation), in patients under mechanical ventilation for at least 24 h

- Patients aged 18 years or more

- The patient must be able to receive either one of the two arms of treatment defined for the study

- Information will be given to the patient and his family and informed consent will be obtained during the first three days: through family if the patient is incapable of giving consent.

- Microorganisms (s) sensitive to the specified antibiotic regimen (ref 5.3)

**6.2 Exclusion criteria**

- Patients do not match the criteria for inclusion

- Patients aged under 18 years of age

- Pregnant patients

- Another infectious outbreak documented the day of the BAL.

- Patients with acquired (blood diseases, HIV,...), induced (immunosuppressive drugs, cancer, radiation therapy) or congenital immunosuppresion.

- Steroids for a period exceeding 15 days.

- Leukopenia (1000 GB/mm) or neutropenia (500 PN/mm)

- Purulent pleural effusion, pulmonary abscess

- Cystic fibrosis

- Patient previously receiving antibiotic treatment according to the following terms:

1. Ongoing curative antibiotic therapy

2. Antibiotics within 3 days before the diagnosis of VAP, excluding surgical antibiotic prophylaxis (defined according to the consensus conference "antibiotic prophylaxis in the surgical environment in adult" December 11, 1992) (27)

3. Use of antibiotics not authorized in the study (see list)

- Allergy to antibiotics used in the study

- Inclusion in another study assessing antibiotic treatment, either the treatment or prevention of nosocomial pulmonary disease

- Refusal to participate

- Lack of informed consent by the patient or his family

**7. Criteria of evaluation of the main objective**

**7.1 Parameters to define the clinical response :** highest temperature over 24 hours for hyperthermia or lowest in case of hypothermia ; leukocytosis (morning sample) character of bronchial secretions ; chest X-ray (radiological score: Appendix II) ; PaO2/FiO2 ratio (in a stable situation, outside intercurrent acute events)

**7.2 Main judgment Criteria**

The main criterion of judgment is the clinical response.

**7.2.1 Failure ;**

- Death for what ever reason during the protocol

- Initial clinical worsening judged after D3 of treatment:

septic shock (Appendix IV) or "sepsis syndrome" (Score of organ failure related to sepsis: SOFA Appendix V) without another documented infection - increase in the score of organ failure (increase in SOFA score by 2 points or more) with identification of the same microorganism at a new BAL

- Persistence or increase of all signs of pulmonary infection after D3 of treatment:

- temperature > 38.5° or < 36 °

- > 12000 WBC / mm^3^ or < 5000 / mm^3^

- purulent bronchial secretions

- absence of pulmonary radiological improvement and persistence of the original microorganism in the BAL

- A need to change the initial antibiotic for treatment intolerance (Appendix VI)

If failure is diagnosed, regardless of the day (After D3 of treatment), the patient is assessed as failure in the final evaluation at D21.

**7.2.2 Cure :**

Assesment will be made at D21, in the final evaluation, using the following criteria

**Intubated patient :**

- Temperature < 38° or > 36.5°
- Leukocytes < 12000 / mm^3^

These 2 first criteria will not be used in the assessment if there is a documented extra-pulmonary infection.

- None purulent tracheal aspiration
- Chest X Ray normal or enhanced from D0 (decrease of the radiological score of at least 2 points)
- PaO_2_/FiO_2_ > 300 mmHg or improvment of more than 20% compared to D16.

**Extubated patient :**

- Temperature < 38° or > 36.5°
- Leukocytes < 12000 / mm^3^

These 2 first criteria will not be used in the assessment if there is a documented extra-pulmonary infection.

- Absence of sputum or non-purulent sputum
- Chest X Ray normal or enhanced from D0 (decrease of the radiological score of at least 2 points)
- Ventilation on air or return to their previous level of oxygen requirement

**Home :**

The patient will be deemed cured at D21 if:

- 1. If data from the last day of hospitalization data is compatible with all the cure criteria
- 2. Absence of a new antibiotic treatment directed towards a lung infection (telephone interview at D21).

**7.3 Intercurrent pulmonary infectious events**

Any intercurrent infectious event is documented by a new BAL. If a different bacteria is present at significant concentration, the patient is considered superinfected and is still evaluable at D21 ; if the BAL is sterile, treatment is continued and the patient will be evaluate at D21

**7.4 Non-pulmonary infectious intercurrents events**

Any modification of antibiotics will be preceded by the collection of bacteriological samples deemed necessary. If an intercurrent non-pulmonary Infection is documented, the patient is considered superinfected and remains evaluable at D21 on intention to treat.

**8 Conduct of test**

**8.1 Pre-inclusion**

Takes place on the day of completion of the BAL if the patient meets the criteria for inclusion (except bacteriological data). A follow-up book is then opened for the patient (daily monitoring sheet: annex VII)

**8.2 Final Inclusion**

The final inclusion will be determined after the bacteriological result of the BAL becomes available. A sheet for final inclusion, including bacteriological information and a copy of the informed consent is faxed to the principal investigator.

**8.3 Randomisation**

Is carried out in the final inclusion. It will be performed by the main investigator centre using a pre-defined centre randomization table. The assigned treatment arm is communicated by fax between 8 a.m. and 12 p.m., Monday to Friday and at the latest by D5.

1. **Methodology**

- **Pre-inclusion**
- verification of the criteria for inclusion (except bacteriological data) and exclusion
- realization of the BAL
- realization of 3 blood cultures
- simplified acute physiologic score: SAPSIIS II (Appendix VIII)(Appendix IX) Mc Cabbe score - score of organ failure (ODIN: Appendix X)
- -score of organ failure related to sepsis (SOFA: Appendix V)
- **Inclusion**
- verification of the bacteriological criteria for inclusion.
- **D0 of treatment**
- SAPS II (Appendix VIII) score
- score of organ failure (ODIN: Appendix X)
- score of organ failure related to sepsis (SOFA: Appendix V)
- **D1 of treatment**
- SAPS II (Appendix VIII) score
- score of organ failure (ODIN: Appendix X)
- score of organl failure related to sepsis (SOFA: Appendix V)
- rectal swabs
- **D4 of treatment**
- **decrease of dosages of beta-lactam - interim evaluation**

**. D6 of Treatment**

- Stop Aminoglycoside

- **D9 of treatment**
- rectal swabs
- interim evaluation of healing
- **D16 of treatment**
- rectal swabs
- interim evaluation of healing
- **D leave intensive care or hospital if < 21 days** : intermediate evaluation of healing

**D21 : Final evaluation = failure or cure**

**For all days preceding the final assessment, patients will be monitored according to clinical and biological criteria. All intercurrent events will be taken into account (lung infections, other infections, shock, toxic manifestations relating to the treatment...) (Appendix VII)**

1. **Legal dispositions**

The test is conducted under the responsibility of the investigator, under the French research regulation.

**9.1 Comité Consultatif de Protection des Personnes se prêtant à la Recherche Biomédicale (CCPPRB) : (Ethical and research committee)**

Before performing the study, the principal investigator will submit the project for the opinion of the CCPPRB. The study will begin only after being deemed favourable and without reservations from the Committee and after applying for insurance. The investigator will inform the CCPPRB of any subsequent amendments and any serious or unexpected intercurrent event occurring during the study that would affect the safety of subjects or the conduct of the study. In this case, information will be issued to all investigators. Each collected informed consent (Appendix XI) will be presented in a sealed envelope.

**9.2 Directors of intensive care unit and Director’s hospital Agreement :**

Their agreement depends on the participation of an investigator centre.

**9.3 Side effects :**

It is the responsibility of investigators to document all intercurrents events occurring during the study. Intercurrents events include drug interactions or organic diseases that emerge during the study, regardless of the relationship with the treatment received. Antibiotics used in the test have known pharmacokinetic characteristics and potential adverse effects. All measures must be taken to define the relationship of causation between the treatment and intercurrent event.

In the event of a death, a statement must be made to the principal investigator who will inform the regulatory agency within 24 hours (from Monday to Friday). Serious events (annex VI) will be the subject of a declaration to the pharmacovigilance centre connected to the investigator centre (Act of 24 May 1984) (Declaration of serious intercurrent event worksheet: Appendix XII). For accountability to the studied treatments, the investigator should inform the lead investigator, who will take care of the regulatory agency information. All treatments adapted to the state of the patient will be implemented to ensure the support of intercurrent event. The continuation of the ongoing antibiotic treatment is left to the discretion of the investigator in charge of the patient.

**10. Secondary objectives**

For each of the two groups the following studies will be conducted:

**10.1 Study of nosocomial superinfections**

**Objective:** to evaluate the influence of the duration of antibiotic treatment on the prevalence of fatal pulmonary and extra-pulmonary infections (definitions of nosocomial infections: Appendix XIII).

**Method:** Consideration of all the infectious intercurrents events observed while the patient is in an intensive care unit, for a maximum period of 3 weeks.

**10.2 Study of the microbiological flora**

Antibiotics can modify original endogenous flora of hospitalized patients. It can promote the growth of exogenous bacteria in these patients. A short course of antibiotics may represent a lower risk of disruption of bacterial flora in hospitalised patietns with equal clinical effectiveness.

**Objective:** study the changes in intestinal flora through pathological specimens (antibiotic resistance phenotype +/-genotypes).

**Method:** the study of the endogenous flora is based on the analysis of faecal flora taken by rectal swabs, on the identification of the species present at the beginning and end of treatment and the determination of their antibiotic susceptibility phenotype.

Identification of the character isogenic strains is to be conducted for some species (Pseudomonas aeruginosa, Klebsiella pneumoniae, Enterobacter aerogenes, Enterobacter cloacae, Staphylococcus aureus and Enterococcus faecalis) found in J0 J8, J15 and variation in sensitivity to antibiotics, as well as for pathogenic species which strains are identical to faecal strains (study of the bacterial Ecology: Appendix XIV).

**10.3 Economical study**

Little data is currently available on the economic impact of the duration of antibiotic therapy. A hypothesis is that the cost of a defined treatment is proportional to its length. However, two factors directly related to the duration of treatment may alter the overall cost of support for the infection (25): the failure of the first line treatment and/or the emergence of resistant bacteria (antibiotic pressure). The additional costs then correspond to the treatment of the infections and/or complications (septic shock...). These additional costs are rarely evaluated (23). It therefore appears necessary to compare the costs of two therapeutic strategies (8 days versus 15 days of antibiotic treatment). Such a study will evaluate the influence of the duration of treatment on the different costs of management of nosocomial pulmonary disease and its complications through analysing the costs of hospitalization, drugs, devices, and other medical expenditures.

**Method** : Study cost-identification, from the point of view of the hospital will be collected from D0 to D21 and the direct costs attributable to nosocomial pneumonia and its complications. Data will be collected and the costs will be analysed for each of the two groups:

**# Hospital fees** : for a nosocomial infection, it is difficult to discern the hospitalization related to infection and that related to the initial pathology. Hospitalization in connection with the lung disease and its complications will be calculated by counting the number of days during which the patient will be treated by anti-infective treatments. The costs will be calculated through the analytical accounting of a centre of reference.

**# Treatments** : will be taken into account for the specific treatment of lung disease, pulmonary and extra-pulmonaires infections and, where appropriate, of septic shock. Costs identified until D21 will include:

- anti-infective (antibacterial and antifungal)

- for septic shock: a Swan-Ganz probe insertion: hemodialysis or Hemofiltration: specific treatments

- management of the complications caused by treatment (e,g, pseudomembranous colitis) these costs will be calculated from the "markets" 1996 price at the centre of reference. For hemodialysis or Hemofiltration, a daily average cost will be calculated by a reference centre.

**# Investigations**: These will be taken into account as the specific investigations related to lung disease and infections, namely:

- radiological examinations (x-rays and scanners)

- bacteriological examinations

- assays of antibiotics.

These costs will be calculated from the nomenclature of the acts in force since 1996.

**11. Statistical design**

**11.1 Experimental design**

**It is a therapeutic trial of equivalence, with two parallel groups of which the main criterion is the rate of clinical cure at the end of treatment (D21).**

**11.2 Number of patients**

The calculation of the number of patients was carried out under the following conditions: the equivalence is accepted up to a maximum variation of 10% between clinical cure rates. The rate of successful treatment for the usual duration of antibiotic therapy (15 days) is between 80 and 90%.

Patients are randomized after the result of the BAL: it is estimated that 20% of patients pre-included may not be included permanently. This produces a risk equal to 0.20. The number of subjects to be included must be a minimum of 275 and maximum of 350 for the initial value of the rate of cure and the risk (chosen, as indicated in the table below):

| %  cured | α | β | n/group | N pre included | Total included |
| --- | --- | --- | --- | --- | --- |
| 0.90 | 0.05 | 0.20 | 110 | 275 | 220 |
| 0.85 | 0.10 | 0.20 | 110 | 275 | 220 |
| 0.80 | 0.10 | 0.20 | 140 | 350 | 280 |

The number of patients selected for the study is 350.

**11.3 Study duration**

With the recruitment of investigators centres, we estimate the total number of patients included per year to be 120. Therefore, a total study duration of 3 years is estimated in order to recruit the required number of patients who will additionally be followed for 21 days. Every 4 months, a balance sheet of the inclusions will be sent to all investigators centres. To be accepted in the final analysis, each centre must recruit at least 6 patients.

- 1. **Statistical analysis**
- Patients excluded from the analysis include those whose bacteriological samples collected during pre-inlusion do not respond to the criteria laid down.
- Analysis: Non-excluded patients will be analysed by the treatment defined by the drawing of lots (intention to treat). Comparatability of the 2 groups will be performed on the main characteristics and prognostic factors with traditional tests: test t Student for quantitative variables and test of Chi 2 qualitative variables. For the main criterion, the equivalence will be judged by the confidence interval of the odds ratio of the percentage risk (test of decentralized Chi 2).

**12. Presentation of the results**

Communication will be presented to the « l’Agence Nationale d’Evaluation des Médicaments » to various scientific societies, society of critical care of French language (SRLF), French society of anesthesia and surgical critical care (SFAR), international conferences and submitted for publication.

**APPENDIX**

**Appendix I : Radiologic Score**

By quadrant: 0: normal

1: interstitial infiltrate

2: non-confluent cellular infiltrate

3: condensation

Total = sum scores of each of the 4 quadrants

*Weinberg et al., Am Rev Respir Dis. 1984 ; 130 : 791.*

**Appendix II : Bronchoalveolar lavage**

1. **Sample**

Patient sedated - paralyzed - positive end expiratory pressure = 0 - FiO2 = 1

Endotracheal aspiration performed before the bronchoscopy – avoid lidocaine flush through the channel of the fiberscope – avoid aspiration of bronchial secretions through the suction channel - injection of room temperature sterile 0.9% saline – BAL performed in the pulmonary segment corresponding to the territory indicated radiologically. In the case of bilateral breach, the BAL will be carried out where purulent secretions are sitting, or if they do not exist in the lower lobes.

- The BAL is achieved with 3 fractions of 40 cc, with aspiration after each instillation. - The first fraction is analyzed separately. It is used only for the research of Legionella, Mycobacteria and fungal infections.

- The other 2 fractions are grouped together in a sterile container and sent to the laboratory (container type propylene, on which cells do not adhere).

**2) Analysis of the sample**

**Methods :** -2 polyvitex chocolate agar bacitracin-1 Sabouraud chloramphenicol agar blood agar plates (box)-1 agar Sabouraud chloramphenicol (tube)

- tubes of distilled water of 10 ml

- Mucomyst (N-acetyl cysteine), diluted to 1/10

- Pasteur pipettes

**reviews made :** - GRAM stain - counting the total number of cells - formula cell GIEMSA staining, established on 300 to 400 elements - search optional for viral or according to the clinical orientation cytopathic effect - inclusion culture: identification and susceptibility testing

- **Direct exam:** From the washing liquid, prepare two samples with the cytospin.
- 1 - Color with reagents of Diff-Quick (2 to 3 drops for filing): acknowledgement of the number of polymorphonuclear leukocytes (classified as: rare; many; very many), and also the presence of ciliated cells, macrophages and cells not identified in bacteriology.
- 2 - the second by Gram stain of color: observing the presence of bacteria.
- **culture**

Centrifuge 10 ml of BAL 10 mn at 4000 RPM. Decant the supernatant so that it remains about 0.5 ml of base add 0.5 ml of diluted Mucomyst 1/10 and shake the Vortex with this dilution, streak the Sabouraud in the tube for the research of Aspergillus and a blood agar for research of Pneumococcus (plater 2 drops) from the previous dilution to make a 1: 100 dilution, 2 drops in 10 ml sterile distilled water. Put 2 drops of this dilution and spread out on each of the media (blood agar and chocolate agar, Sabouraud agar). Put the blood and chocolate agar under CO_2_ and 37 ° C; the Sabourauds were incubated at 30 ° C.

- results

After 24 and 48 hours, look at the plates, quantify and identify each species. 1 colony "dilute" agar: 1.103 /ml

- interpretation

- Positive threshold : 10^4^ CFU/ml

**Appendix III : Severity Criteria implying to start antibiotics after the BAL**

- **Respiratory failure:** at least one of the following criteria: 1) PaO^2^ / FiO^2^ < 200 mmHg; 2) score radio >3

- **Cardiovascular failure:** at least one of the following criteria: 1) Systolic Arterial Pressure < 60 mmHg; 2) use of vasopressor or inotropic substances (Dopamine > 5 µg / kg / min) to keep a SAP >90mmHg ; 3) volume expansion >1.5 litres / 24 hours

- **Renal failure:** diuresis <30 ml/h or 0.5 ml/kg/h or 6 H diuresis <200 ml

- **Haematological failure:** at least one of the following criteria: 1) leukocytosis <2 000 / mm^3^; 2) platelets <40 000 / mm^3^

- Patient with a prosthetic valve (known or documented)

**Appendix IV : Definition of septic shock**

- Systolic blood pressure < 90 mm Hg (or decrease from the usual value by at least 30 mmHg not responding to volume expansion, in the presence of a septic syndrome.

*Baumgartner J.D., Critical Care Medecine 1992 ; 20 (7) : 953-960.*

**Appendix V : Sepsis-related Organ Failure Assessment (SOFA)**

**Appendix VI : Complications related to treatment**

- Acute renal failure (2X serum creatinine from the value before treatment):-severe: anuria - conserved diuresis
- Skin complications:-severe: Lyell syndrome – other skin lesions
- Digestive complications:-severe: pseudomembranous colitis – other
- Hematological complications:-severe: anemia, leukopenia, neutropenia, thrombocytopenia grade 3 or 4 (table below)-other ranks
- Allergic complications:-severe: anaphylactic shock – other

Others (hepatitis, enkephalopathy, lung disease)Haematologic toxic acute and subacute ( WHO)

| haematological | grade 0 | grade 1 | grade 2 | grade 3 | grade 4 |
| --- | --- | --- | --- | --- | --- |
| Haemoglobin G/100ml | **11** | **9.5-10.9** | **8.0-9.4** | **6.5-7.9** | **6.5** |
| leucocytes G/L | **4.0** | **3.0-3.9** | **2.0-2.9** | **1.0-1.9** | **1** |
| Neutrophils G/L | **2.0** | **1.5-1.9** | **1.0-1.4** | **0.5-0.9** | **0.5** |
| Platelets G/L | **100** | **75-99** | **50-74** | **25-49** | **25** |
| Haemorrhage | **No** | **purpura** | **small** | **large** | **severe** |

**Appendix VII : Daily follow up sheet**

**Day :**

**Alive**  **Dead**

**ICU** : Yes No

**Transfert** : Ward

Home

**Evolution of the early onset VAP :**

Temp max

Leucocytosis

PaO_2_ /FIO_2_

Radiological score

ODIN (Appendix X)

SOFA (Appendix V)

Shock ( Annexe IV ) : Yes No

Surperinfection : Yes No

BAL result:

**Secondary infections :**

***Sites* : -** Bacteremia Yes No Antibiotic sensitivity

- Catheter Yes No

- Urines Yes No

- Other :

**Economic data :**

***Examinations* : -** Ultrasound

- Chest X-Ray

- CT scan

***Equipment :***  - Swan Ganz catheter

- hemodialysis

- hemofiltration

***Treatment :*** *-* Antibiotics

- Other

**Side effects of the treatment** :

Present : Yes No

Relationship with the treatment Yes No

**Appendix VIII : SAPS II**

**Appendix IX : Mc Cabbe Classification**

This classification takes into account the existence of underlying disease and the appreciation of its prognosis in the three months preceding hospitalization and resuscitation. This very simple grouping allows us to distinguish between very different prognosis groups.

- 0: absence of underlying disease or underlying diseases not affecting the vital prognosis
- 1: underlying diseases are affecting the overall prognosis within 5 years
- 2: fatal – death from underlying diseases estimated within 1 year

**Appendix X : Definition of organ failure ( ODIN )**

Respiratory failure (at least one of the following criteria):

- PaO^2^ < 60mmHg with FiO^2^ = 0, 21

- Artificial ventilation

Cardiovascular failure (at least one of the following criteria in the absence of hypovolemia):

- systolic blood pressure <90 mmHg with signs of peripheral hypoperfusion
- use of inotrope or vasopressor to maintain a systolic blood pressure >90 mmHg

Renal failure (at least one of the following criteria in the absence of chronic renal failure):

- serum creatinine >300µmol/l
- diuresis <500 ml/24 hours or 180 ml / 8 h
- need of renal replacement therapy

Neurological failure (at least one of the following criteria):

- score of Glasgow <6 (in the absence of sedation)
- sudden onset of confusion

Hepatic failure (at least one of the following criteria):

- bilirubin >100µmol/l
- phosphatase alkaline (x 3 normal)

Hematological failure (at least one of the following criteria):

- Hematocrit <20%
- leukocytosis <2,000 / mm^3^
- platelets <40,000 / mm^3^

*Fagon et coll, Intens.Care Med. 1993 ; 19 : 137-144.*

**Appendix XI A : Information for the patient**

Dear Sir,

During your hospital stay, you have developed a pulmonary infection. Samples are carried out under fiberoptic to confirm the diagnosis and to identify the responsible bacteria.

The antibiotic treatment you receive will take account of these microorganisms. The duration of this antibiotic treatment remains unclear but must meet 2 goals: treat the initial infection, and avoid complications.

We propose that you participate in a study that compares two lengths of antibiotic treatment: 8 days or 15 days. The choice of this period of treatment is randomly allocated . All of your other treatment will not be changed by this Protocol. You will benefit from the complementary examinations necessary for your support without additional invasive examination.

The final evaluation will take place 21 days after the start of your treatment. If you are not in hospital by this date, you will be contacted by telephone for us to complete your file. The data collected will be analyzed by computer and strict anonymity is ensured.

**Appendix XI B : Information to store in the study file**

Dear Sir,

During your hospital lenght of stay, you developed a pulmonary infection. Samples are carried out under fiberoptic to confirm the diagnosis and to identify the responsible bacteria.

The antibiotic treatment you receive will take account of these microorganisms. The duration of this antibiotic treatment remains unclear but must meet 2 goals: treat the initial infection, and avoid complications.

We propose that you participate in a study that compares two lengths of antibiotic treatment: 8 days or 15 days. The choice of this period of treatment is randomly allocated . All of your other treatment will not be changed by this Protocol. You will benefit from the complementary examinations necessary for your support without additional invasive examination.

The final evaluation will take place 21 days after the start of your treatment. If you are not in hospital by this date, you will be contacted by telephone for us to complete your file. The data collected will be analyzed by computer and strict anonymity is ensured.

Patient signature :

(or family member )

Relationship :

**Appendix XI C : Agreement to enroll the study**  :  «**Essai randomisé évaluant la durée de traitement des pneumopathies nosocomiales précoces sous ventilation : 8 ou 15 jours »**

I undersigned, (name, first name)..................... possible relationship (with the patient) ....have received information concerning this Protocol. The study focuses on the comparison of two periods of antibiotic treatment for pulmonary infection. The final assessment takes place on the 21st day and may require a phone call at my home. Under the Act of 20 December 1988, to protect the people that lend themselves to biomedical research, this study received the favourable opinion of the « comite de protection des personnes se pretant a la recherche biomedicale » (CPPRB) of Franche-Comté. The University Hospital of Besançon, promoter of the study, has contracted insurance under the terms of the Act of 20 December 1988. The data collected will be analyzed by computer, ensuring anonymity. Under the computer and freedom law, you can at any time exercise your right of access and correction.

I understand that I can stop my participation in this study at any time without this changing the quality of care.

If you agree to participate in this study, we ask you to sign this consent.

I declare having been informed by Dr.... of the nature and conduct of this study and I agree to participate.

Place ................. Date..........................199..

Investigator signature Patient signature

(or relatives if impossible for the patient)

**Appendix XII : Side effects declaration**

Mail to : Dr Capellier Service de réanimation médicale CHU Besançon

Fax : 03 81 66 90 13

Centre : Postal Code:

Investigator :

Patient :

Randomization: ARM: Short Long

Treatment : Onset After BAL result

Starting Treatment Date :

Side effects Date :

Description :

Complications :

Relationship with the treatment : Yes No

If Yes which arguments

Treatment : Stop

Pursuit

Adjustment of the dosage

**Appendix XIII : Nosocomial Superinfection definitions**

- Bacteremia: 1 positive blood culture or 2 positive blood cultures if Staphylococcus épidermidis
- catheter: cultures of the tip of the catheter > 10 3 cfu /ml
- Urine : cultures >10 5 cfu / ml and Neutrophils > 10 / field
- Pulmonary: BAL sample culture >10 4 ufc/ml
- Sinusitis: sinus puncture positive
- Surgical wounds: growth of bacteria on local sample

**Appendix XIV : Study of bacteriological ecology changes (performed in some centers)**

In some centres, stool sampling is performed for each patient on the first day of treatment, and then at D8-D15. In addition, all bacteriological samples are retained.

Cultures. The rectal swab will be seeded on selective media (media of Chapman, Slanetz, Drigalski and agar Hyper-Agar). Usual bacteriological samples are treated according to standard protocols.

- Identification and antibiotics sensitivity phenotypes. For rectal specimens, all strains will be identified. Only the following species will be kept for 1 month: Pseudomonas aeruginosa, Enterobacter cloacae, Enterobacter aerogenes, Klebsiella pneumoniae, Staphylococcus aureus and Enterococcus faecalis. It will be performed a sensitivity by the method of diffusion agar (disk Pasteur TM, Kirby-Bauer method). The phenotype of resistance will be carried over to the following table:

Resistance Phenotype of bacteria isolated from rectal and pathological phenotype

| **Bacteria** | **Ps.aeruginosa** | **K.pneumoniae** | **E.aerogenes** | **E.cloacae** | **S.aureus** | **E.faecalis** |
| --- | --- | --- | --- | --- | --- | --- |
|  |  |  |  |  |  |  |
| Wild |  |  |  |  |  |  |
|  |  |  |  |  |  |  |
| Penicillinase |  |  |  |  |  |  |
|  |  |  |  |  |  |  |
| EBLS |  |  |  |  |  |  |
|  |  |  |  |  |  |  |
| Inducible Kephalosporinase |  |  |  |  |  |  |
|  |  |  |  |  |  |  |
|  |  |  |  |  |  |  |
| D2 |  |  |  |  |  |  |
|  |  |  |  |  |  |  |
| Meti-R |  |  |  |  |  |  |
|  |  |  |  |  |  |  |
| High level R |  |  |  |  |  |  |
| Kana-Genta |  |  |  |  |  |  |
|  |  |  |  |  |  |  |
| High level R. |  |  |  |  |  |  |
| Kana |  |  |  |  |  |  |

**Conservation of strains for genotyping:** rectal strains, all strains of the same species with a variation in sensitivity to antibiotics over time will be kept. For pathogenic strains, will be kept strains belonging to the species found in the stool. Strains will be kept in agar conservation.

**Identification of the character isogenic:** this part will be carried out only by the laboratory of senior investigator centre (CHU of Besançon). Couples (or more) strains defined above will be sent with their sensitivity. Will be typed up 1000 strains. Clonal identification will be carried out by determination of profiles of macro-restriction of total DNA by (Chief method) pulsed field gel electrophoresis. The clonal relationships study will be conducted using the GelComparR (Applied MathTM): calculation of the Pearson correlation coefficient. Staphylococcus aureus NCTC 8325 digested by SmaI is introduced all 3 or 4 wells for the standardization of gels and inter-gels comparisons. Restriction enzymes are: SmaI for strains of Staphylococcus and Enterococcus strains, XbaI for strains of Enterobacteriaceae and DraI for Gram-negative bacilli aerobic strict.

**References**

1- A’Court C.D., Garrard C.S.

Nosocomial pneumonia in the ICU : new perspectives on current controversies

In : Vincent J.L.

Yearbook of intensive care and emergency medicine

Springer 1995 : 726-47.

2- Bradbury F.

Comparaison of azithromycin versus clarithromycin in the treatment of patients with lower respiratory tract infection

J. Antimicrob. Chemother. 1993 ; 31 (suppl E) : 153-62.

3- Bodenham A., Shelly M.P., Park G.R.

The altered pharmacokinetics and pharmacodynamics of drugs commonly used in critically ill patients

Clin. Pharm. 1988 ; 14 : 347-73.

4- Chan R., Hemeryck L., O’Regan M., Clancy L., Feely J.

Oral versus intravenous antibiotics for community acquired lower respiratory tract infection in a general hospital : open, randomised, controlled trial

BMJ 1995 ; 310 : 1360-3.

5- Chastre J., Fagon J.Y., Trouillet J.L.

Diagnosis and treatment of nosocomial pneumonia in the patients in intensive care units

Clin Infect Dis 1995 ; 21 (suppl.3) : 226s-37s.

6- Cometta A., Baumgartner J.D., Lew D., Zimmerli W., Pittet D. et al.

Prospective randomized comparaison of imepenem monotherapy with imepenem plus netilmicin for treatment of severe infections in nonneutropenic patients

Antimicrob Agents and Chemother 1994 ; 38 (6) : 1309-13.

7- Craven D.E., Steger K.A., Barat L.M., Duncan R.A.

Nosocomial pneumonia : epidemiology and infection control

Int Care Med 1992 ; 18 : 3-9.

8- Fagon J.Y., Stephan F., Novara A.

Epidémiologie des pneumopathies acquises sous ventilation artificielle

In : Chastre J., Fagon J.Y.

Pneumopathies nosocomiales et ventilation artificielle

Paris : Masson, 1995 : 1-12.

9- Fagon J.Y., Chastre J., Hance A.J., Montravers P., Novara A. et al.

Nosocomial pneumonia in ventilated patients : a cohort study evaluating attributable mortality and hospital stay

Am J Med 1993 ; 94 (2) : 281-8.

10- Fink M.P., Snydman D.R., Niederman M.S., Leeper K.V., Johnson R.H.

Treatment of severe pneumonia in hospitalized patients : results of a multicenter, randomized, double-blind trial comparing intravenous ciprofloxacin with imepenem-cilastatin

Antimicrob Agents and Chemother 1994 ; 38 (3) : 547-57.

11- Galvez-Vargas R., Bueno-Cavillas A., Garcia-Martin M.

Epidemiology, therapy and costs of nosocomial infection

PharmacoEconomics 1995 ; 7 (2) : 128-40.

12- Garner J.S., Jarvis W.R., Emori T.G., Horan T.C., Hugues J.M.

CDC definitions for nosocomial infections

Am J Inf Control 1988 ; 16 : 128-40.

13- Garrait V., Crémieux A.C., Carbon C.

Bases pharmacologiques (pharmacocinétiques et pharmacodynamiques) des traitements antibiotiques courts

In : Carbon C., Pocidalo J.J., Cremieux A.C.

Traitements antibiotiques courts

Paris : Arnette Blakwell, 1995 : 27-32.

14- Gilbert D.N.

Once-daily aminoglycoside therapy

Antimicrob. Agents Chemother. 1991, 35 (3) : 399-405.

15- Joshi N., Localio A.R., Hamory B.H.

A predictive risk index for nosocomial pneumonia in the intensive care unit

Am J Med 1992 ; 93 (2) : 135-42.

16- Kappstein I., Schulgen G., Beyer U., Geiger K., Schumacher M. et al.

Prolongation of hospital stay and extra costs due to ventilator-associated pneumonia in an intensive care unit

Eur. J. Clin. Microbiol. Infect. Dis. 1992 ; 11 : 504-8.

17- Kemper C.A., Deresinski S.C.

Diagnosis and management of pneumonia

Pharmacotherapy 1991 ; 11 (suppl. 2) : 84s-89s.

18- La Force F.M.

Systemic antimicrobial therapy of nosocomial pneumonia : monotherapy versus combination therapy

Eur. J. Clin. Microbiol. Infect. Dis. 1989 ; 8 (1) : 61-8.

19- Marrie T.J.

Community-acquired pneumonia

Clinical infectious diseases. 1994 ; 18 : 501-515.

20- Parker S.E., Davey P.J.

Practicalities of once-daily aminoglycoside dosing

J. Antimicrob. Chemother. 1993, 31 : 4-8.

21- Petitjean O., Prevot M., Lortholary O., Tod M., Nicolas P.

Pharmacocinétique comparée des aminosides utilisés en dose unique journalière

Med. Mal. Inf. 1993 ; 23 : 23-36.

22- Petitpretz P.

Traitement court des pneumopathies communautaires

In : Carbon C., Pocidalo J.J., Cremieux A.C.

Traitements antibiotiques courts

Paris : Arnette Blakwell, 1995 : 81-7.

23- Pittet D.

Les infections nosocomiales

Med. et Hyg. 1995, 53 : 1687-9.

24- Prod’hom G., Leuenberger P., Koerfer J., Blum A., Chiolero R. et al.

Nosocomial pneumonia in mechanically ventilated patients receveing antacid, ranitidine, or sucralfate as prophylaxis for stress ulcer

Ann of Intern Med 1994 ; 120 : 653-62.

25- Rho J.P., Yoshikawa T.T.

the cost of inappropriate use of anti-infective agents in older patients

Drugs and Aging 1995 ; 6 (4) : 263-7.

26 - Schlemmer B., Garrouste M. T., Le Gall J.R.

Traitement antibiotique des pneumopathies nosocomiales , bases de réflexion et attitude pratique

In : Réanimation et Médecine d’Urgence 1989, 288-301.

27- Société Française d’Anesthésie et Réanimation

Conférence de consensus : antibioprophylaxie en milieu chirurgical chez l’adulte

Paris : 10 et 11 Décembre 1992.

28- Ter Braak E., De Vries P., Bouter K.P., Van Der Vegt S.G., Dorrestein G.C. et al.

Once-daily dosing regimen for aminoglycoside plus β-lactam combination therapy of serious bacterial infections : comparative trial with netilmicin plus ceftriaxone

Am. J. Med. 1990, 89 : 58-66.

29- Tulkens P.M.

Pharmacokinetic and toxicological evaluation of once-daily regimen versus conventional schedules of netilmicin and amikacin

J. Antimicrob. Chemother. 1991, 27 (suppl C) : 49-61.

30- Unertl K.E., Lenhart F.P., Forst H., Peter K.

Systemic antibiotic treatment of nosocomial pneumonia

Int Care Med 1992 ; 18 : 28-34.

31- Wade W.E., McCall C.Y.

Pharmacist-managed aminoglycoside therapy in combination with β-lactam agent in the treatment of nosocomial pneumonia in critically ill patients

Pharmacotherapy 1995 ; 15 (2) : 216-20.
